# Association between early sonographic findings and acetabular index at the age of 6 months: a prospective observational study

**DOI:** 10.1186/s12887-022-03268-4

**Published:** 2022-04-26

**Authors:** Wen-Chieh Chang, Kuei-Hsiang Hsu, Yu-Ping Su

**Affiliations:** 1 Department of Orthopaedics and Traumatology, Taipei Veterans General Hospital, Taipei, Taiwan; 2 Department of Orthopaedics, School of Medicine, National Yang Ming Chiao Tung University, Taipei, Taiwan

**Keywords:** Developmental dysplasia of the hip, Graf method, Femoral head coverage ratio, Acetabular index

## Abstract

**Background:**

This study investigated the association between early Graf classification and femoral head coverage (FHC) with the acetabular index (AI) at the age of 6 months.

**Methods:**

A prospective observational study was conducted between 2017–2018. Patients requiring Pavlik harness treatment and patients with syndromic dislocation or neurogenic dislocation were excluded. In total, 169 newborns with the first ultrasound performed at the mean age of 12.3 (0–15) days, the second ultrasound performed at the mean age of 3.2 (2.5–4.1) months, and the AI measured at the age of 6.6 (4.3–7.1) months were enrolled. The correlation between the AI and first and second alpha angles and FHC measurements, and the correlation of dysplasia in early ultrasound with dysplasia in the AI were analyzed.

**Results:**

At the first ultrasound, only the FHC (*P* = .02) demonstrated a significant negative correlation with the AI. At the second ultrasound, both the alpha angle (*P* < .01) and FHC (*P* < .01) demonstrated a significant negative correlation with the AI. With the AI as a reference, the sensitivity, specificity, positive predictive value (PPV), and negative predictive value (NPV) were found to be 77%, 7%, 5%, and 81%, respectively, for the first Graf; 91%, 37%, 9%, and 98%, respectively, for the first FHC measurement; 82%, 90%, 35%, and 99%, respectively, for the second Graf; and 95%, 97%, 68% and 99%, respectively, for the second FHC measurement.

**Conclusions:**

FHC and alpha angle exhibited significant negative correlations with the AI at six months, both ultrasound parameters may have the potential to predict AI in DDH screening. Compared to the ultrasound measurements taken at 2 weeks, Graf and FHC at 3 months demonstrated superior sensitivity, specificity, PPV, and NPV to detect abnormal AI. The best timing to perform ultrasound examination may need further research.

## Background

Developmental dysplasia of the hip (DDH) refers to an abnormal development of osseous and soft tissue structures on the hip joint, which results in a spectrum of diseases ranging from mild dysplasia to hip dislocation. The reported incidence is 3 to 5 out of 1000 newborns [1, 2].

Universal physical examination for hip stability followed by selective ultrasound screening for newborns with risk factors for DDH (ie, infants delivered in breech presentation, family history, and history of clinical instability) is generally used as the screening strategy in infants under 4 months old [3, 4]. The Graf method and femoral head coverage (FHC) ratio are the two most widely used techniques for ultrasound interpretation; both methods provide simple and quantitative results for accurate diagnosis [5]. After the age of 4 months, as the ossification of the femoral head begins, the acetabular index (AI) in the anteroposterior view of a pelvic radiograph becomes effective in assessing hip morphology.

The correlation between ultrasound and the AI has been discussed in the literature [6–12]. However, the results have varied because of the multiple study designs. Ultrasound is highly operator-dependent, and inconsistencies among observers may have contributed to the disparate results obtained in previous studies. Different timings of examination for mild hip dysplasia as it matured may also have affected correlations between ultrasound and radiograph findings. Mild dysplasia and mild instability noted in the first few weeks of life in newborns are generally considered likely to have a benign course, with up to 88% of cases resolving by 8 weeks of age [13, 14]. However, few studies have discussed the correlation of the Graf method and FHC at different ages with AI in consecutive patients, and whether interpretation of the ultrasound at different ages affects the correlation with the AI measurement remains unclear. Furthermore, limited evidence exists regarding the association of initial dysplasia manifested using the Graf method or FHC at different ages with dysplasia defined through the AI; the reliability of using ultrasound to predict an abnormal AI remains uncertain.

The primary purpose of this study was to investigate the correlation of the alpha angle in the Graf method and the FHC ratio at different ages with the AI measured at the age of 6 months. The secondary purpose was to evaluate the association of dysplasia identified through ultrasound at various ages with the dysplasia measured using the AI at 6 months.

## Methods

A prospective observational study was conducted between January 1, 2017, and December 31, 2018. Newborns delivered in our institute and newborns referred for DDH were included. Patients with neurogenic dislocation, syndromic dislocation, and patients receiving Pavlik harness treatment were excluded from the final analysis. The baseline patient characteristics were documented using medical records. The study was approved by the local institutional review board.

All newborns underwent the Barlow and Ortolani test [15, 16] and a survey of risk factors for DDH after delivery. Patients considered to have risk factors were female, twins, firstborn, delivered in breech presentation, or had a positive family history. Hip stability was classified as stable, subluxatable, and dislocatable or dislocated according to the Barlow and Ortolani test results. Stable hips were defined as hips whose hip center remained static during the Barlow and Ortolani examination; subluxatable hips were defined as any laxity of the hip center without dislocation; dislocatable or dislocated hips were defined as hip center completely displaced from the acetabulum. Subluxatable hips were conformed following the agreement of 2 senior pediatric orthopedic surgeons.

Patients were classified into the following 3 categories according to their risk factors and Barlow and Ortolani test results: stable hips with risk factors; subluxatable, dislocatable or dislocated hips with or without risk factors; and stable hips without risk factors. Patients in the stable hips with risk factors and the subluxatable, dislocatable or dislocated hips groups underwent ultrasound examination for bilateral hips after informed consent was obtained from legal guardian of participants. The first ultrasound was conducted immediately after the neonatal hip physical examination and risk factors survey. The second ultrasound was performed at an age of approximately 3 months to evaluate the hip development.

A senior pediatric orthopedic surgeon with musculoskeletal ultrasound certification performed the hip ultrasound. Three repetitive ultrasound examinations were performed at the bilateral hips for the enrolled newborns. A valid ultrasound image was one that contained the lower iliac margin at the triradiate cartilage, the chondroosseous border of the proximal femur, the labrum, and the deepest point of the acetabulum [17]. The most representative image of each hip was selected and interpreted using the Graf method and FHC ratio. The ultrasonography device was equipped with a 7.5-MHz linear transducer (LOGIQ e ultrasound, GE Healthcare, USA). The transducer was placed vertically on the hip joint. Infants were placed in the lateral decubitus position with the hips slightly flexed, adducted, and internally rotated while undergoing ultrasound examination [17].

**Fig. 1:**
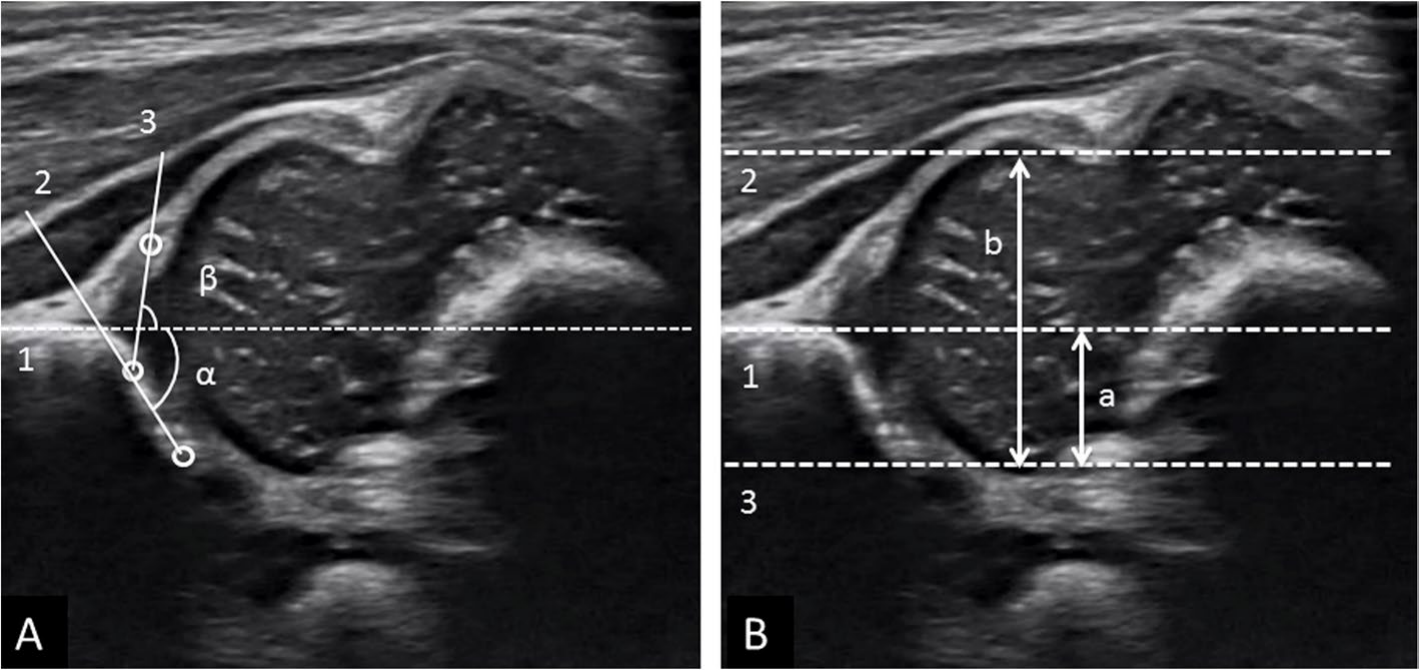
**A** Graf method. 1. Base line. 2. Bony roof line. 3. Cartilaginous roof line. **B** Femoral head coverage ratio; measured as a/b. 1. Base line. 2. Line parallel to base line and connected to the most lateral femoral head. 3. Line parallel to base line and connected to the most medial femoral head

In the Graf method, the alpha angle refers to the angle between the vertical cortex of the ilium (base line) and the bony acetabular roof line; the beta angle is formed by the base line and the triangular labral fibrocartilage line (Fig. 1). Graf type I was defined as an alpha angle ≥ 60°; type IIa and IIb were defined as an alpha angle between 50°and 59° in newborns younger or older than 3 months old, respectively; type IIc and type D were defined as an alpha angle between 43°and 49° with a beta angle greater or less than 77°, respectively; type III and IV were dislocated hips defined as an alpha angle < 43° with an absence or presence of inverted labrum, respectively. Graf type > I was defined as immature [17]. The FHC ratio was measured as the length from the base line to the parallel line connected to the most medial femoral head divided by the length between the 2 lines parallel to the base line that connected to the most medial and lateral femoral head (Fig. 1). FHC < 50% was considered abnormal [18–20].

**Fig. 2:**
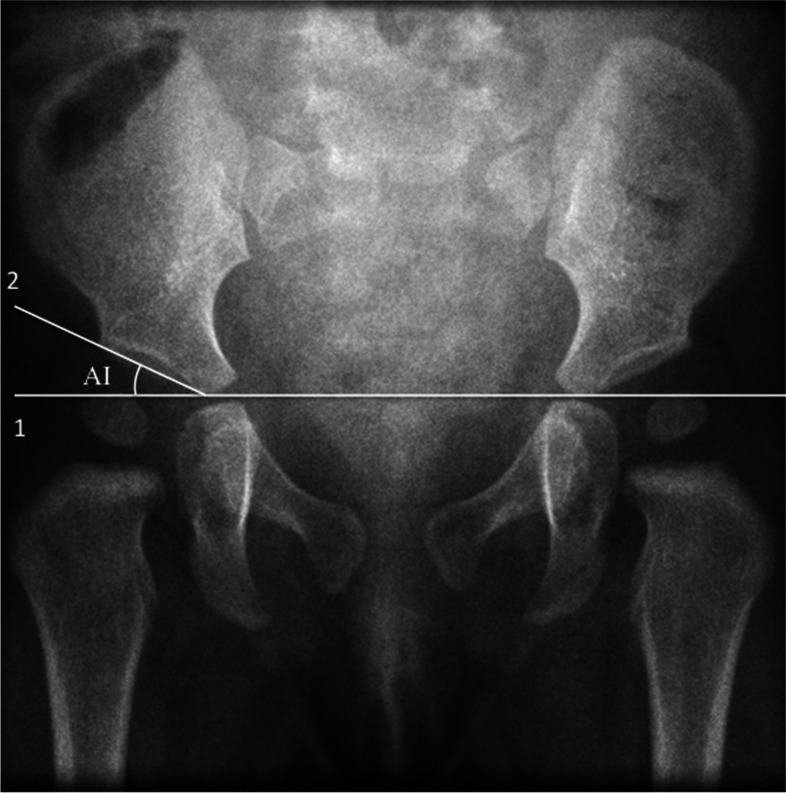
Acetabular index (AI) formed by Hilgenreiner line (Line1) and a line drawn from the triradiate epiphysis to the lateral edge of the acetabulum (Line 2)

An anteroposterior pelvic radiograph for patients was taken at the age of 6 months. The AI is the angle formed by the Hilgenreiner line and a line drawn from the triradiate epiphysis to the lateral edge of the acetabulum (Fig. 2). An AI > 23.5° was considered abnormal in infants at the age of 6 months [21].

All ultrasound parameters were measured by an attending pediatric orthopedic physician. The AI was measured by an attending pediatric orthopedic physician who was blinded to the ultrasound results. To calculate intraobserver variability, 50 hips were measured using the Graf method, the FHC ratio, and the AI. The observer blinded to the previous results interpretated these measurements a second time 1 week after they were taken. The intraclass correlation coefficients for the alpha angle, beta angle, FHC and AI measurement were 0.92 (95% CI: 0.80-0.99), 0.88 (95% CI: 0.80-0.94), 0.95 (95% CI: 0.92-0.97), and 0.97 (95% CI: 0.92-0.99), respectively.

Images from patients who completed the first and second ultrasound examinations and the radiograph taken of patients at 6 months old were analyzed. The primary outcome was the correlation of the first and second alpha angles and the FHC with the AI at 6 months; the secondary outcome was the sensitivity, specificity, positive predictive value (PPV), and negative predictive value (NPV) of the first and second Graf classification and FHC ratio in detecting an abnormal AI at 6 months.

### Statistical analysis

The associations of alpha angle and FHC with AI were assumed to be linear [10, 12]. Linear regression analysis was used to assess the association of alpha angle and FHC at the first and second ultrasound examinations with measurement of the AI at 6 months. A chi-square test was used to investigate the correlation of abnormal Graf classification and FHC ratio with an abnormal AI. The sensitivity, specificity, PPV, and NPV of the first and second Graf and FHC were calculated using the AI measurement at the age of 6 months as a reference. Significance was defined as *P* < 0.05. Calculations were performed using SPSS version 22 (IBM Corp. Released 2013. Armonk, NY, USA).

## Results

**Fig. 3:**
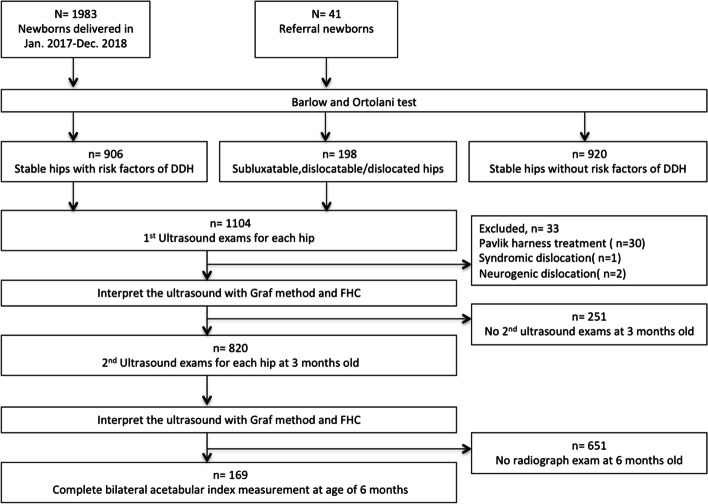
Flowchart of patients inclusion and exclusion in this study

From January 1, 2017, to December 31, 2018, a total of 1983 newborns were delivered in our institute and 41 referral newborns were included in our study (Fig. 3). All newborns underwent Barlow and Ortolani test and screening for risk factors of DDH. Totally 1104 newborns underwent their first ultrasound examination of the bilateral hips, including 906 newborns with stable hips but a presence of risk factors as well as 198 newborns with either subluxatable, dislocatable, or dislocated hips. Thirty-three newborns were excluded from the final analysis, including 30 patients undergoing Pavlik harness treatment, 1 patient with syndromic dislocation, and 2 patients with neurogenic dislocation. After the first examination, 251 newborns did not underwent the second hip ultrasound at age of 3 months due to clinically stable hips with normal ultrasound at the first examination; 651 newborns did not underwent pelvic radiograph at age of 6 months because of stable hips with normal ultrasound at the second examination. In total, 820 newborns underwent a second ultrasound and 169 infants underwent the radiograph at the age of 6 months. Images from 169 newborns with completed first and second ultrasounds and completed radiograph were analyzed (Fig. 3).

Among these 169 newborns, the mean age at the first and second ultrasound exam and at the radiograph was 12.3 (0–15) days, 3.2 (2.5–4.1) months, and 6.6 (4.3–7.1) months, respectively. The characteristics of enrolled newborns were summarized in Table 1.

**Table 1:** Patient characteristics

Characteristics, *n* = 169	
Age at 1^st^ ultrasound (days)	12.3 (0–15)
Age at 2^nd^ ultrasound (months)	3.2(2.5–4.1)
Age at radiograph (months)	6.6 (4.3–7.1)
Female	95 (56%)
Gestational age (weeks)	38.3 (26–41)
Birth weight (gram)	2948.2 (1418–4218)
Twins	8 (5%)
Firstborn	63 (37%)
Breech presentation	23 (14%)

**Fig. 4:**
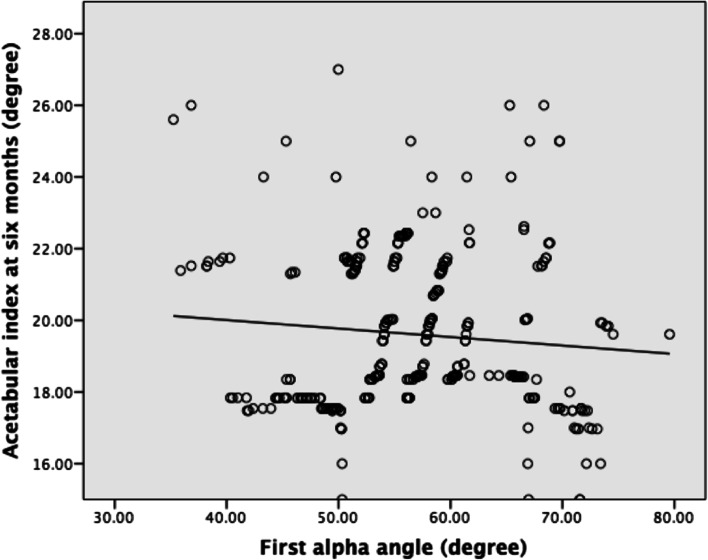
Linear correlation of 1^st^ alpha angle and acetabular index at 6 months

**Fig. 5:**
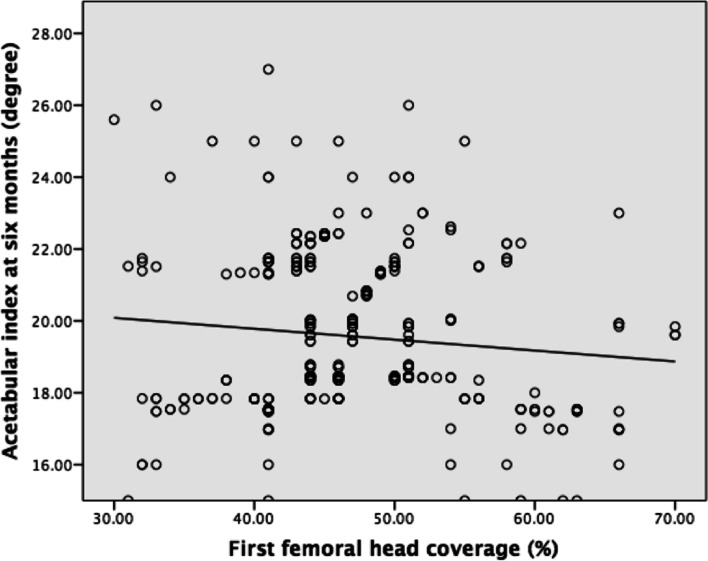
Linear correlation of 1^st^ femoral head coverage and acetabular index at 6 months

At the first ultrasound, the mean alpha angle was 57° (35°-79°) and the mean FHC ratio was 47% (30%-70%). Both the alpha angle and FHC ratio at the first ultrasound demonstrated a negative correlation with the AI (Figs. 4 & 5). In the alpha angle, the regression equation was F(1,336) = 2.977 (*P* = 0.09), with R^2^ of 0.009. The predicted AI was equal to 20.955 − 0.024 (alpha angle) degree. In the FHC ratio, the regression equation was F(1,336) = 5.396 (*P* = 0.02), with R^2^ of 0.016. The predicted AI was equal to 21.246 − 0.024 (FHC ratio) degree, where FHC is presented as a percentage.

**Fig. 6:**
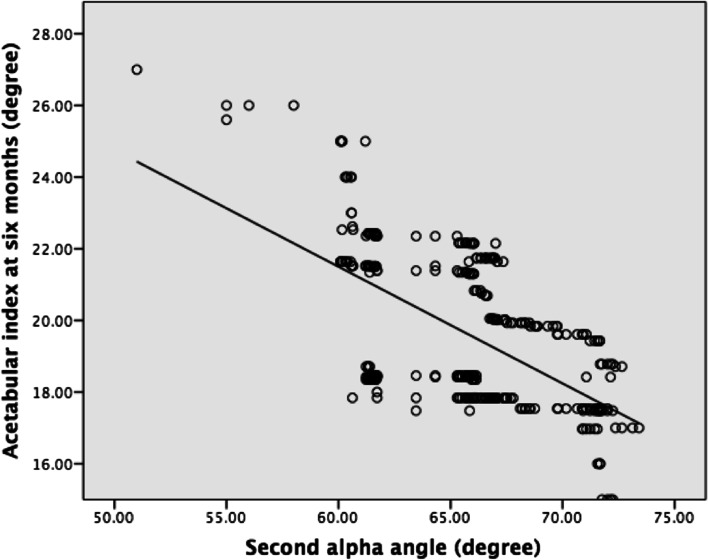
Linear correlation of 2^nd^ alpha angle and acetabular index at 6 months

**Fig. 7:**
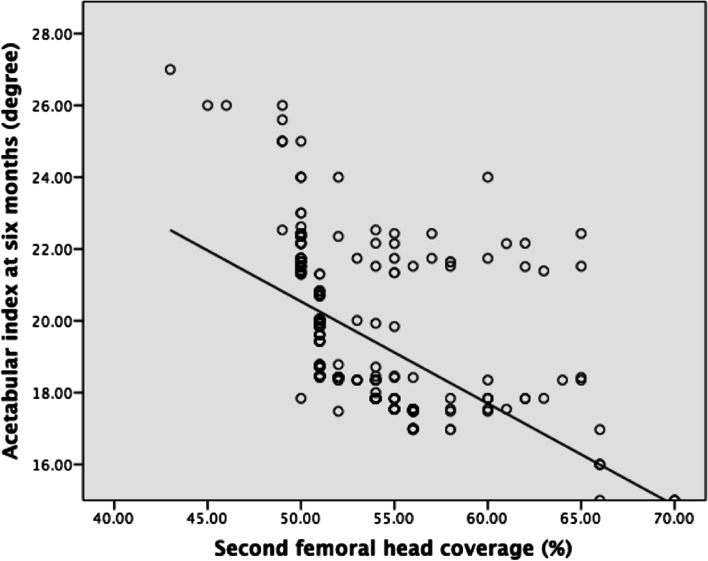
Linear correlation of 2^nd^ femoral head coverage and acetabular index at 6 months

At the second ultrasound examination, the mean alpha angle was 66° (51°-72°), and the mean FHC ratio was 53% (43%-70%). Both the alpha angle and FHC demonstrated a significant negative correlation with the AI (Figs. 6 & 7). The regression equation for the alpha angle was F(1,336) = 182.83 (*P* < 0.01), with R^2^ of 0.352. The predicted AI was equal to 41.052 − 0.326 (alpha angle) degree. The regression equation for the FHC ratio was F(1,336) = 136.572 (*P* < 0.01), with R^2^ of 0.289. The predicted AI was equal to 34.741 − 0.284 (FHC ratio) degree, where FHC is presented as percentage.

Regarding the association of dysplasia indicated in the first ultrasound with the dysplasia measured through the AI at 6 months, with the AI at 6 months as a reference, the sensitivity, specificity, PPV, and NPV were found to be 77% (95% CI: 55%-92%), 7% (95% CI: 4%-10%), 5% (95% CI: 4%-7%) and 81% (95% CI: 65%-91%), respectively, for the first Graf classification and 91% (95% CI: 71%-99%), 37% (95% CI: 31%-42%), 9% (95% CI: 8%-10%), and 98% (95% CI: 94%-100%), respectively, for the first measurement of FHC (Table 2).

**Table 2:** Association of 1^st^ ultrasound with AI at age of six months in 338 hips

	Graf method	FHC
Normal 1^st^ ultrasound (with normal /abnormal AI at 6 months)	27 (22/5)	118 (116/2)
Abnormal ultrasound (with normal /abnormal AI at 6 months)	311 (294/17)	220 (200/20)
Sensitivity^a^ (95%CI)	77% (55–92%)	91% (71–99%)
Specificity^a^ (95%CI)	7% (4–10%)	37% (31–42%)
Positive predictive value^a^ (95%CI)	5% (4–7%)	9% (8–10%)
Negative predictive value^a^ (95%CI)	81% (65–91%)	98% (94–100%)

*AI* acetabular index, *FHC* femoral head coverage, *CI* confidence interval

^a^ Using acetabular index at age of 6 months as reference

At the second ultrasound examination, using the AI at 6 months as a reference, the sensitivity, specificity, PPV, and NPV were found to be 82% (95% CI: 60%-95%), 90% (95% CI: 86%-93%), 35% (95% CI: 27%-44%), and 99% (95% CI: 97%-99%), respectively, for the second Graf classification and 95% (95% CI: 77%-100%), 97% (95% CI: 94%-98%), 68% (95% CI: 53%-80%), and 99% (95% CI: 98%-100%), respectively, for the second FHC measurement (Table 3).

**Table 3:** Association of 2^nd^ ultrasound with AI at age of six months in 338 hips

	Graf method	FHC
Normal 2^nd^ ultrasound (with normal /abnormal AI at 6 months)	287 (283/4)	307 (306/1)
Abnormal 2^nd^ ultrasound (with normal /abnormal AI at 6 months)	51 (33/18)	31 (10/21)
Sensitivity^a^ (95%CI)	82% (60–95%)	95% (77–100%)
Specificity^a^ (95%CI)	90% (86–93%)	97% (94–98%)
Positive predictive value^a^ (95%CI)	35% (27–44%)	68% (53–80%)
Negative predictive value^a^ (95%CI)	99% (97–99%)	99% (98–100%)

*AI* acetabular index, *FHC* femoral head coverage, *CI* confidence interval

^a^ Using acetabular index at age of 6 months as reference

## Discussion

In this study, FHC measured at 2 weeks and alpha angle and FHC measured at 3 months demonstrated a significant negative correlation with the AI at 6 months. The Graf classification and FHC measured at 3 months were superior in sensitivity, specificity, PPV, and NPV compared with the ultrasound taken at 2 weeks by using the AI as reference to diagnose hip dysplasia.

The correlation between FHC, alpha angle and AI has been studied in the literature with various results. This is likely due to different settings employed by ultrasound operators, different image observers, and the timing of the ultrasound and radiograph examinations. However, most evidence support that both FHC and alpha angle are negatively correlated with the AI. Copuroglu et al. [10] investigated the correlation of the alpha angle with the AI measured at nearly the same time in 33 infants. All 7 observers reported a negative correlation between the alpha angle and the AI. Spaans et al. [12] reported that both the alpha angle and FHC measurements had a significant negative correlation with the AI. However, only a weak correlation was observed between the ultrasound and the AI in diagnosing abnormal hip morphology. Kitay et al. [22] performed hip ultrasound and pelvic radiograph in 31 infants at approximately 6 months of age. They concluded that FHC was negatively correlated with the AI, while no association was found between alpha angle and AI. The relatively low interobserver reliability for alpha angle measurement may contribute to the insignificant relationship between alpha angle and AI in their study.

The discrepancy between the ultrasound and radiograph in diagnosing hip dysplasia has been extensively studied. Whether the abnormal ultrasound can predict abnormal AI is still controversial. Dornacher et al. [8] investigated the correlation between ultrasound performed at the mean age of 7 weeks and radiograph obtained at the mean age of 15 months in 90 children; they found that, although all dysplastic hips achieved normal Graf morphology at follow-up, 32.8% and 29.4% of hips exhibited mild and severe residual dysplasia at the time of radiological assessment, respectively. No significant correlation was observed between the initial Graf classification and the AI at the time of radiological follow-up. They, therefore, recommended a radiological follow-up for all treated hips. Atalar et al., [11] in a study involving 44 infants with a mean age 21.7 weeks, reported that radiography had a sensitivity of 61% and a specificity of 87% in detecting DDH with the Graf classification as a reference. They concluded that low-grade dysplasia in ultrasound may be overlooked by radiography. Results regarding the correlation between the ultrasound performed at different age with the radiograph are relatively limited in previous studies. Pillai et al. [9] analyzed the correlation between the Graf classification interpreted at the age of 0 to 28 days, 29 to 77 days, and > 77 days with radiography performed at 6 months in 71 infants. The researchers used the AI at 6 months as a reference, and ultrasound performed at the age of 0 to 28 days, 29 to 77 days, and > 77 days yielded an accuracy of 55%, 82%, and 94%, respectively, and a specificity of 56%, 83%, and 96%, respectively. These results are consistent with our findings of specificity and accuracy increasing with age. Tan et al. [23] compared the hip ultrasound findings in the first three months with the pelvic radiograph at 1 year in 160 patients. They suggested that both FHC and Graf classification showed close correlations, with no statistically significant differences, when compared to the acetabular index at 1 year. However, they reported that both ultrasound parameters had 100% negative predictive value for identifying an abnormal femoral head position on radiographs performed at 1 year.

In the present study, ultrasound performed at 3 months not only exhibited a stronger correlation with the AI compared with the ultrasound performed at 2 weeks but also had a more favorable sensitivity, specificity, PPV, and NPV. This may be attributed to the improved delineation of landmarks for the alpha angle and FHC measurements as the hip matured. This finding is also consistent with the natural history of DDH, in which most mild dysplasia or instability noted in the first few weeks of life spontaneously resolve.

This study is unique in that it reports the relationship between the Graf method and FHC measurements at different ages with the AI in consecutive patients, an approach that is rare in previous studies. This study also provides evidence for the natural history of hip maturation. This study used a single ultrasound operator and a single observer to eliminate interrater variability, which may have been a confounding factor in previous study designs. Our study had several limitations. First, the sample size was relatively small, and the duration of the follow-up was short. Second, although the intraobserver variability was low, it may still affect the results of the interpretation of the ultrasound. Third, it may be difficult to have single sonographer or image observer in the real clinical setting, which was designed in our study. The impact of interobserver variability and whether our results could be practically applied to DDH screening may need further study. Finally, a dynamic ultrasound was not performed in this study, which may have affected the interpretation of the ultrasound. The role of the initial ultrasound in predisposing radiography, as well its prognostic value in clinical utility, require further investigation with a larger sample size and longer follow-up.

## Conclusions

FHC and alpha angle exhibited significant negative correlations with the AI at six months, both ultrasound parameters may have the potential to predict AI in DDH screening. Compared to the ultrasound measurements taken at 2 weeks, Graf and FHC at 3 months demonstrated superior sensitivity, specificity, PPV, and NPV to detect abnormal AI. Further researches are required to determine the best timing for ultrasound examination.

## Data Availability

The datasets used and/or analyzed during the current study available from the corresponding author on reasonable request. The datasets generated in the study can be deposited publically.

## Abbreviations

AI: Acetabular index
CI: Confidence interval
DDH: Developmental dysplasia of the hip
FHC: Femoral head coverage
NPV: Negative predictive value
PPV: Positive predictive value
